# Effects of practice on visuo-spatial attention in a wayfinding task

**DOI:** 10.1007/s00426-020-01463-5

**Published:** 2021-01-20

**Authors:** Mai Geisen, Kyungwan Kim, Stefanie Klatt, Otmar Bock

**Affiliations:** 1 Institute of Exercise Training and Sport Informatics, German Sport University Cologne, Cologne, Germany; 2 Institute of Sports Science, University of Rostock, Rostock, Germany

## Abstract

Several studies have evaluated the distribution of visuo-spatial attention in a wayfinding task, using gaze direction as an indicator for the locus of attention. We extended that work by evaluating how visuo-spatial attention is modified by wayfinding practice. Young and older participants followed prescribed routes through a virtual city on six trials. Each trial was followed by a route recall test, where participants saw screenshots of intersections encountered, and had to indicate which way to proceed. Behavioral and gaze data were registered in those tests. Wayfinding accuracy increased from trial to trial, more so in young than in older persons. Total gaze time, mean fixation time, and the vertical scatter of fixations decreased from trial to trial, similarly in young and older persons. The horizontal scatter of fixations did not differ between trials and age groups. The incidence of fixations on the subsequently chosen side also did not differ between trials, but it increased in older age. We interpret these findings as evidence that as wayfinding practice increased, participants gradually narrowed their attentional focus to the most relevant screenshot area, processed information within this focus more efficiently, reduced the total time in which attention dwelled on the rejected side of the screenshot, but maintained the total time on the chosen side. These dynamic changes of visuo-spatial attention were comparable in young and older participants. However, it appears that decision-making differed between age groups: older persons’ attention dwelled longer on the chosen side before they made their choice.

## Introduction

Finding one’s way in an unfamiliar building or city can be quite challenging. A number of studies have quantified the cognitive demand of wayfinding with the dual-task approach. They observed substantial interference between a wayfinding task and another, concurrent task (Lindberg and Gärling, 1982), particularly when the concurrent task required visuo-spatial processing (Garden et al., 2002; Meilinger et al., 2008), and when participants approached an intersection (Allen and Kirasic, 2003; Hartmeyer et al., 2017; Hilton et al., 2020). In accordance with established dual-task reasoning, these results indicate that wayfinding competes with the other task for cognitive resources, mainly for resources related to visuo-spatial processing, and mainly at times when participants must decide which way to proceed across an intersection.

Another group of studies evaluated the cognitive demand of wayfinding by registering gaze position, an indicator for the locus of visuo-spatial attention. In a learning phase, participants were passively guided through a virtual environment or were shown a sequence of static images from such an environment. In the subsequent test phase, they saw a sequence of static images that displayed some of the intersections encountered in the learning phase, and had to indicate in which direction to proceed. Authors found that in the test phase, the *percentage of correct direction choices* ranged between 49 and 85%, (de Condappa and Wiener, 2016; Hartmeyer et al., 2017; Hilton et al., 2020; Wiener et al., 2011), and that it was higher when the correct choice was to walk straight on rather than to make a turn (Hartmeyer et al., 2017). The *horizontal scatter* of gaze fixations was larger than the *vertical scatter*, and varied from intersection to intersection (Wiener et al., 2012). This suggests that the relevant information for direction choices was broadly distributed along the horizontal dimension. Shortly before participants chose the direction to proceed, they directed their gaze mainly at locations which were relevant for wayfinding (de Condappa and Wiener, 2016; Grzeschik et al., 2019): they preferably looked to the left if they subsequently chose to turn left, and they preferably looked to the right if they subsequently chose to turn right. Specifically, the *incidence of fixations on the chosen side* began to increase above chance level at about 800–500 ms before participants made their choice, and reached a plateau of about 75–90% shortly before they reported their choice (Wiener et al., 2011, 2012).

Several studies have presented not just one learning phase followed by one test phase, but rather scheduled an interleaved sequence of learning and test phases. This allowed authors to evaluate the effects of practice by monitoring how variables of interest change from one learning-phase-test-phase pair to the next, or in short, from one trial to the next. The *percentage of correct direction choices* increased significantly from trial to trial (de Condappa and Wiener, 2016; Hartmeyer et al., 2017; Hilton et al., 2020; Wiener et al., 2011). The time that participants looked at an intersection before making a choice, *total gaze time*, decreased from trial to trial (Wiener et al., 2011). The average duration of individual fixations, *mean fixation time,* did not change significantly between trials (Hilton et al., 2020). Likewise, the *incidence of fixations on the chosen side* did not change significantly between trials (Hilton et al., 2020). Importantly, however, the absence of trial-to-trial changes should not be interpreted as evidence that the gaze pattern is rigid and unsusceptible to practice: above studies either implemented just two (Hilton et al., 2020) or three trials (Hartmeyer et al., 2017), which possibly was not enough to substantiate effects of practice, or they implemented up to six trials, but did not evaluate trial-to-trial changes (i.e., they did not include a factor “trial” in their statistical analyses). One purpose of the present study therefore was to evaluate gaze behavior over six wayfinding trials and to include the factor “trial” in our analyses.

Wayfinding abilities are known to decline in healthy aging (Head and Isom, 2010; Moffat, 2009). Previous studies have found that the *percentage of correct direction choices* was lower in older participants than in young ones (Grzeschik et al., 2019; Hartmeyer et al., 2017; Hilton et al., 2020), but increased from trial to trial similarly in both age groups (Hartmeyer et al., 2017; Hilton et al., 2020). *Total gaze time* was higher in older persons than in young ones (Grzeschik et al., 2019). *Mean fixation time* and the *incidence of fixations on the chosen side* were similar in both age groups, and both parameters did not change from trial to trial in either age group (Hilton et al., 2020). The paucity of significant age effects and the absence of significant age * trial interactions could again be attributable to insufficient practice time: one study implemented 2 trials (Hilton et al., 2020), another one 3 trials (Hartmeyer et al., 2017), and a third 1–5 trials (Grzeschik et al., 2019). A second purpose of our study was therefore to enhance our knowledge about age-related changes by including a group of healthy older persons in our experimental design.

Another gap in our knowledge pertains to the relationship between wayfinding performance and gaze parameters. We are aware of only one study that addressed this issue: the *percentage of correct direction choices* was significantly associated with the the *scatter of fixations* across two trials, and to the *incidence of fixations on the chosen side* on the second, but not on the first trial (Hilton et al., 2020). This study provided a first insight into the relationship between gaze and wayfinding; however, it left open whether practice-related changes of wayfinding performance are associated with practice-related changes of gaze behavior. To close this gap, we quantified the trial-to-trial change in the *percentage of correct direction choices* and correlated it with the trial-to-trial change in gaze parameters.

## Methods

### Participants and questionnaires

Twenty-two young persons (25.14 ± 2.1 years of age, 15 females, 7 males) and 26 older persons (64.38 ± 3.5 years of age, 11 females, 15 males) participated in this study. 20 young and 18 older persons had a university-entrance qualification (“Abitur”). Participants were free of neurological and psychiatric diseases per self-report. Those who wore seeing aids in their daily life continued to use them during the experiment. For the older age group, inclusion criteria were a visus of ≥ 0.8 according to the procedures by Wesemann (2002) and a score of < 20 s on the Timed up and Go Test according to the procedures of Thomas and Lane (2005). We did not screen for cognitive impairment in our older participants, to keep total testing time as short as possible. However, we asked them beforehand, via telephone or e-mail, whether they feel physically and mentally healthy. We reasoned that persons who came at the agreed-upon time to the agreed-upon location without assistance, and who correctly followed all our instructions for a range of quite complex tests, are not likely to suffer from a cognitive impairment that would be detectable by a screening test such as MMSE. Data from five young and seven older participants were not evaluated: these persons either discontinued the experiment because of motion sickness, or they did not produce analysable gaze data. Approval was obtained from the lead institution’s ethics board. All participants signed a written informed consent before testing began.

Participants completed a single testing session, which lasted about 2 h. We first administered three questionnaires. A *demographics* questionnaire enquired participants’ age, sex, and education level. A *wayfinding self-efficacy* questionnaire assessed participants’ confidence for wayfinding even under difficult circumstances; it was based on the Self-efficacy Scale (Beierlein et al., 2013), except that words referring to generic confidence were replaced by words relating to wayfinding confidence. The Self-efficacy Scale consists of three items, each to be responded on a five-point scale ranging from 0 = “does not apply at all” to 4 = “applies fully”; the outcome measure is the sum of scores across all items. A *mobility* questionnaire enquired whether within the last 6 months, participants had driven a car and/or had navigated in an unknown environment. Responses to either item were on a three-point scale with the levels ‘never’, ‘occasionally’ and ‘several times per week’. After completing the questionnaires, participants underwent six trials of a route-finding task, interleaved with six trials of a route recall test.

### Route-finding task

**Fig. 1:**
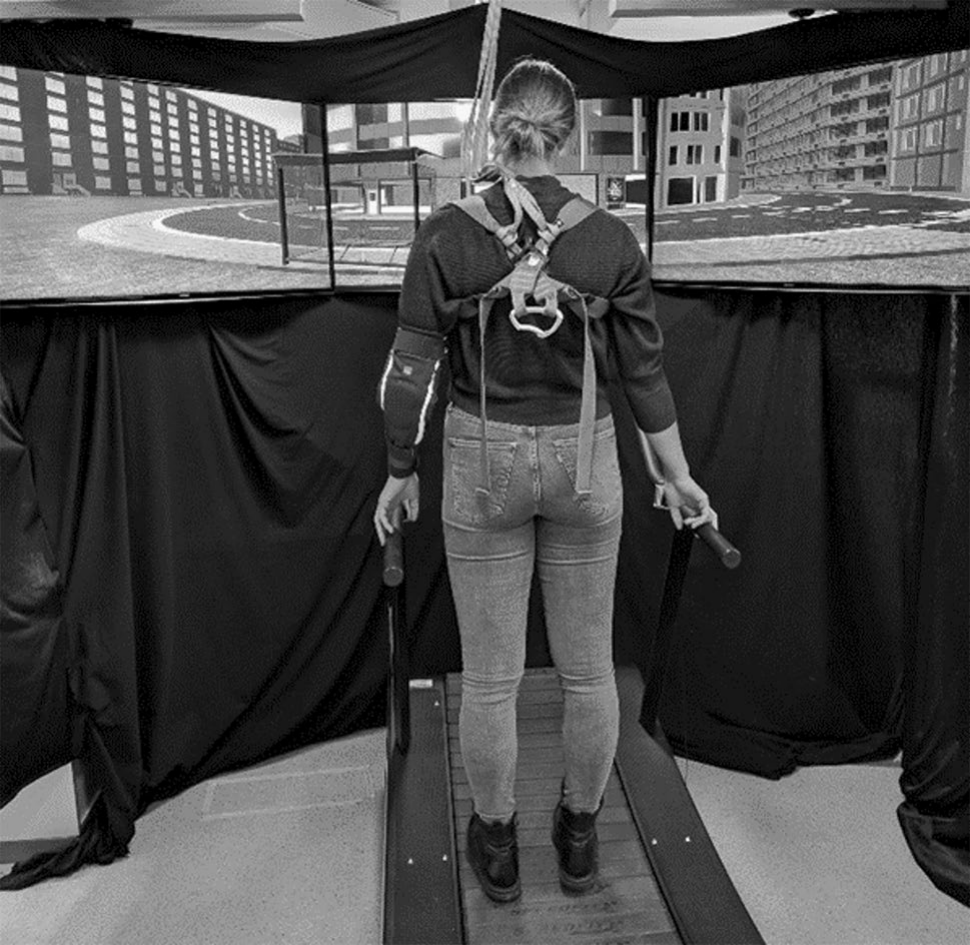
Setup for the route-finding task. The picture shows a participant on the treadmill, secured by a safety harness, who watches the virtual city on three TV screens

Participants mounted a non-motorized treadmill (Speedfit 1000, Vibrafit) on which they could walk at their self-determined speed, protected by a safety harness (see Fig. 1). They faced three 46’’ TV screens, located at eye level. One screen was aligned with the participants’ medio-sagittal plane, and the other two screens were placed to the left and right at an angle of 106°. Taken together, the three screens displayed a 160° view of a computer-generated, urban environment (‚virtual city‘), developed by a commercial provider with the Unity™ game engine. The virtual city consisted of streets with sidewalks, a range of building types, a park, and realistic props such as bus stops, garbage bins, and parked bicycles. Participants progressed forward through the virtual city by walking on the treadmill, and they turned left or right by depressing a switch attached to the left and right handrail of the treadmill, respectively.

**Fig. 2:**
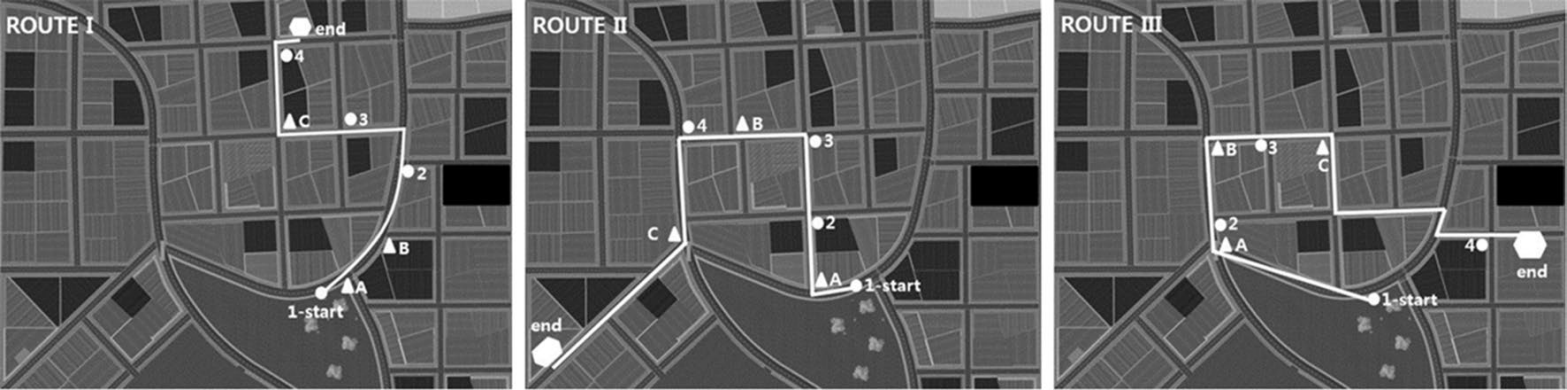
Top views of the three routes. All routes start at the park and end either at a bakery (route I), a bookshop (route II), or a red building (route III). Letters A, B, C denote the positions of screenshots for the landmark sequence test, and numbers 1, 2, 3, 4 denote the positions of screenshots for the route recall test

Participants were asked to walk through the virtual city along three prescribed routes, as illustrated in Fig. 2. All three routes started at the same location in the city park. Participants first walked along route I until they reached the destination, a bookshop. They were then teleported back to the common starting position, and walked along route II towards a bakery. They were then teleported back again to the common starting position, and walked along route III towards a distinctive red building. This completed one trial, i.e., on each trial of the route-finding task, participants walked once on route I, once on route II and once on route III. Participants were asked to walk each route on the right sidewalk.

During the first trial, participants were guided by the experimenter: as they approached an intersection, they received verbal instructions on whether to proceed straight on, turn left, or turn right. During the following five trials, participants decided on their own: as they approached an intersection, they verbally indicated which direction they intended to proceed, and the experimenter corrected them if necessary. This ensured that participants always took the correct direction.

### Route recall test

After completing a trial of the route-finding task, i.e., after walking each of the three routes once, participants dismounted the treadmill. They were then seated in front of a 17” computer monitor with a built-in eye-tracking system (T60, Tobii AB, Danderyd, Sweden). That system registers gaze position at a rate of 60 Hz, and is accurate to within 0.5°; error due to head motion is less than 1° if the head is within a 44 × 22 × 30 cm volume centred 70 cm in front of the screen. To minimize eye-tracking artefacts by ambient light, the setup was shielded by black curtains.

The computer monitor displayed a sequence of 12 screenshots. Each showed one of the intersections that participants had passed while walking on the treadmill, from the same vantage point and in the same order as seen on the treadmill (cf. lettered positions in Fig. 2). Participants first saw a message telling them that the subsequent screenshots will come from the first route, they then saw a sequence of four screenshots from that route, next came a message that the subsequent screenshots will come from the second route, etc., until the last screenshot from the third route had been displayed. Participants were asked to verbally report in which direction the route continued across this intersection, and to press a key which triggered the display of the next intersection. They were encouraged to respond accurately rather than quickly, and they received no feedback about the correctness of their response. After the 12th response, the trial was completed and participants returned to the treadmill for the next trial of the route-finding task.

**Fig. 3:**
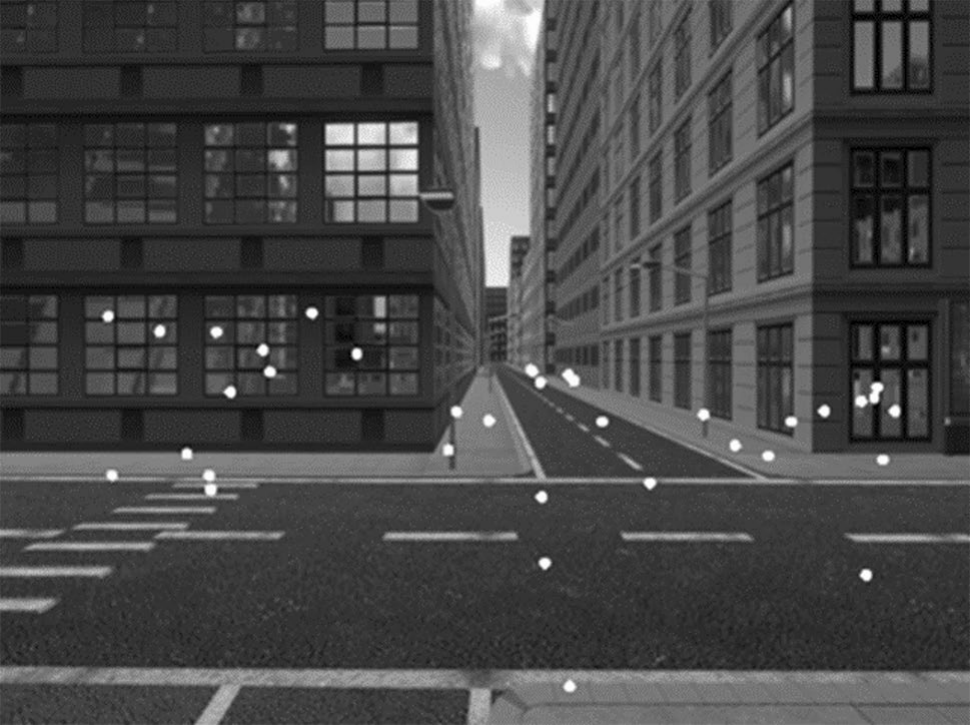
Screenshot of the virtual city, overlaid with fixations. Fixation data are from one participant on one trial. Note that the horizontal scatter of fixations seems to form three clusters, located at the origins of the left-hand street, the street straight ahead, and the right-hand street, respectively

Performance on each trial was quantified as:

- *percentage of correct direction choices* (PercCorDir), as tallied by the experimenter,
- *total gaze time* (TotGazeT): interval from screenshot appearance to the participant’s keystroke, averaged across the 12 screenshots,
- *mean fixation time* (MeanFixT): mean duration of individual fixations, averaged across the 12 screenshots,
- *horizontal scatter of fixations* (HorScatFix): interquartile range of the horizontal component of fixation positions, averaged across the 12 screenshots (interquartile ranges rather than standard deviations were used, since data distributions often were distinctly non-normal, cf. Fig. 3),
- *vertical scatter of fixations* (VertScatFix): same as HorScatFix, but for the vertical component, and
- *incidence of fixations on the chosen side* (IncidFixChosen): number of fixations on the side that participants subsequently chose, relative to the total number of fixations; calculation and statistical analysis of this parameter is quite intricate, and is therefore deferred to the Appendix.

TotGazeT, MeanFixT, HorScatFix, and VertScatFix scores could not be calculated for some screenshot × trial × participant cells, because gaze registration was occasionally disrupted by eye blinks. These missing data were imputed according to the procedure of Bingham et al., (1998), which is superior to a simple substitution by means. Although imputation generally deflates data variance, this undesirable side-effect was attenuated in the present study, since data were averaged across screenshots, i.e., imputed scores were averaged with registered scores (see above). We nevertheless decided to account for deflated variance by setting the significance level for TotGazeT, MeanFixT, HorScatFix, and VertScatFix to *p* < 0.01 rather than the usual *p* < 0.05.

### Visuo-spatial tests

After finishing all six interleaved route-finding and route recall trials, participants completed three additional tests. The Direction Sequence Test was derived from Jansen-Osmann et al., (2007). Participants were told how many direction changes had occurred on the first route, and were then asked to draw that route from memory on a blank sheet of paper. This procedure was repeated for the second, and then for the third route. We subsequently determined, separately for each route, the number of correctly drawn direction changes before the first incorrectly drawn direction change. The sum of those numbers across all three routes served as the outcome of the Direction Sequence Test.

The subsequent Landmark Sequence Test was modelled after Ishikawa and Montello (2006), except that in our study, landmarks were scenes rather than solitary objects. Screenshots of three intersections from the first route were displayed simultaneously on the monitor; these intersections have not been shown on the preceding route recall test (cf. numbered positions in Fig. 2a). Participants were asked to indicate the order in which they had encountered these intersections along the route. The same procedure was repeated for the second, and then for the third route (cf. numbered positions in Fig. 2b and c). The number of correctly ordered intersections, summed up across all three routes, served as the outcome of the Landmark Sequence Test.

The Visual Patterns Test was part of the free software application ‘Psych Lab 101’ (version 2.0.7; Neurobehavioral Systems, Inc.). Visual Patterns Test is modelled after the test of short-term visual memory designed by Corsi (1973). Participants saw a grid of red and blue squares, displayed on the touch screen of a tablet PC. After 2 s, the colour changed, such that all squares now were blue, and participants were asked to tap all squares that previously had been red. A new grid then appeared, etc. The test began with a 3 × 3 grid, and grid size increased on every second trial until the participant gave two wrong responses in sequence. The number of correctly completed trials served as the outcome of the Visual Patterns Test.

### Data analysis

Responses on Self-efficacy Scale, Direction Sequence Test, Landmark Sequence Test, and Visual Patterns Test were compared between both age groups by *t* tests. Responses on the ordinal-scaled mobility questionnaire were compared between age groups by Mann–Whitney *U* tests. For route recall accuracy, we calculated an analysis of variance with the dependent variable PercCorDir, the grouping factor Age (young, older), and the within-group factor Trial (1,…, 6). For gaze behavior, we calculated a multivariate general linear model with the dependent variables TotGazeT, MeanFixT, HorScatFix, and VertScatFix, the grouping factor Age, and the within-group factor Trial. Lack of sphericity was corrected by Greenhouse–Geisser adjustments to the degrees of freedom. Significant effects were followed up by Bonferroni post hoc tests.

To determine whether final performance on the route recall task was related to final knowledge about direction sequences and/or landmark sequences, we calculated a multiple linear regression with the dependent variable ‘PercCorDir on the last trial’, and with the predictors Direction Sequence Test and Landmark Sequence Test. We added the predictor ‘calendric age’, to control for ostensible correlations due to age-related data clustering.

Our remaining analyses dealt with trial-to-trial changes of route recall performance. As a first step, we quantified those changes by calculating, for each participant, the linear regression:

1 $${\text{PercCorDir }} = \, a \, + \, b \, * \, {\text {`trial number'}}.$$

Parameter *b* represents the regression slope: a high positive value indicates that route recall performance improved quickly from trial to trial, and a low positive value indicates that recall performance improved slowly from trial to trial. In what follows, we will refer to this slope as Slope-PercCorDir.

As a second step, we calculated the linear regression slopes for our gaze parameters to yield Slope-TotGazeT, Slope-MeanFixT, Slope-HorScatFix, and Slope-VertScatFix. High negative values indicate that times and scatter decreased quickly from trial to trial, and low negative values indicate that they decreased slowly from trial to trial.

As a third step, we evaluated whether the regression slopes of route recall performance were associated with regression slopes of gaze parameters. To this end, we calculated a (univariate) general linear model with the dependent variable Slope-PercCorDir, and with the predictors Slope-TotGazeT, Slope-MeanFixT, Slope-HorScatFix, Slope-VertScatFix, and ‘calendric age’. Again, ‘calendric age’ was added to control for age-related data clustering. To protect the degrees of freedom, we used a stepwise backward elimination procedure: at each step, the predictor with the highest *p*-score was excluded, until only predictors with *p* < 0.05 remained. If fast trial-to-trial increase of route recall performance was associated with fast trial-to-trial decrease of gaze parameters, then the above model should yield significant predictor effects.

To determine whether trial-to-trial changes of route recall performance were associated with any of the other potential predictors registered in our study, we calculated a general linear model with the dependent variable Slope-PercCorDir, and with the predictors Sex, Self-efficacy Scale, Visual Patterns Test, Mobility I (car use), Mobility II (unknown environment), and ‘calendric age’. Once more, ‘calendric age’ was included to control for age-related data clustering, and a stepwise backward elimination procedure was used to protect the degrees of freedom.

Post hoc power analysis with G*power (Faul et al., 2007) was conducted for the main analysis of interest in our study, the general linear model with Slope-PercCorDir as dependent variable and the gaze parameter slopes as predictors. Using *f*^2^ = 0.15, *α* = 0.05, *n* = 36, number of tested predictors = 1, number of predictors = 6, we yielded 1–*ß* = 0.613 for *R*^2^ increase. We therefore cannot be confident about the non-significance of any given predictor.

## Results

Significant differences between age groups emerged for Visual Patterns Test (*t*(34) =  − 5.134; *p* < 0.001; *ŋ*^2^ = 0.437), Direction Sequence Test (*t*(34) =  − 3.955; *p* < 0.001; *ŋ*^2^ = 0.315), and Landmark Sequence Test (*t*(34) = − 2.602; *p* < 0.05; *ŋ*^2^ = 0.166) as young persons performed better than older ones, and for mobility I (*Z*_corrected_ = 3.224; *p* < 0.01; *ŋ*^2^ = 0.229), as young persons used cars less often than older ones. No age differences were observed for Self-efficacy Scale (*t*(34) = 0.855; *p* > 0.05 *ŋ*^2^ = 0.021) and for mobility II (Z_corrected_ = − 1.73; *p* > 0.05; *ŋ*^2^ = 0.088).

**Fig. 4:**
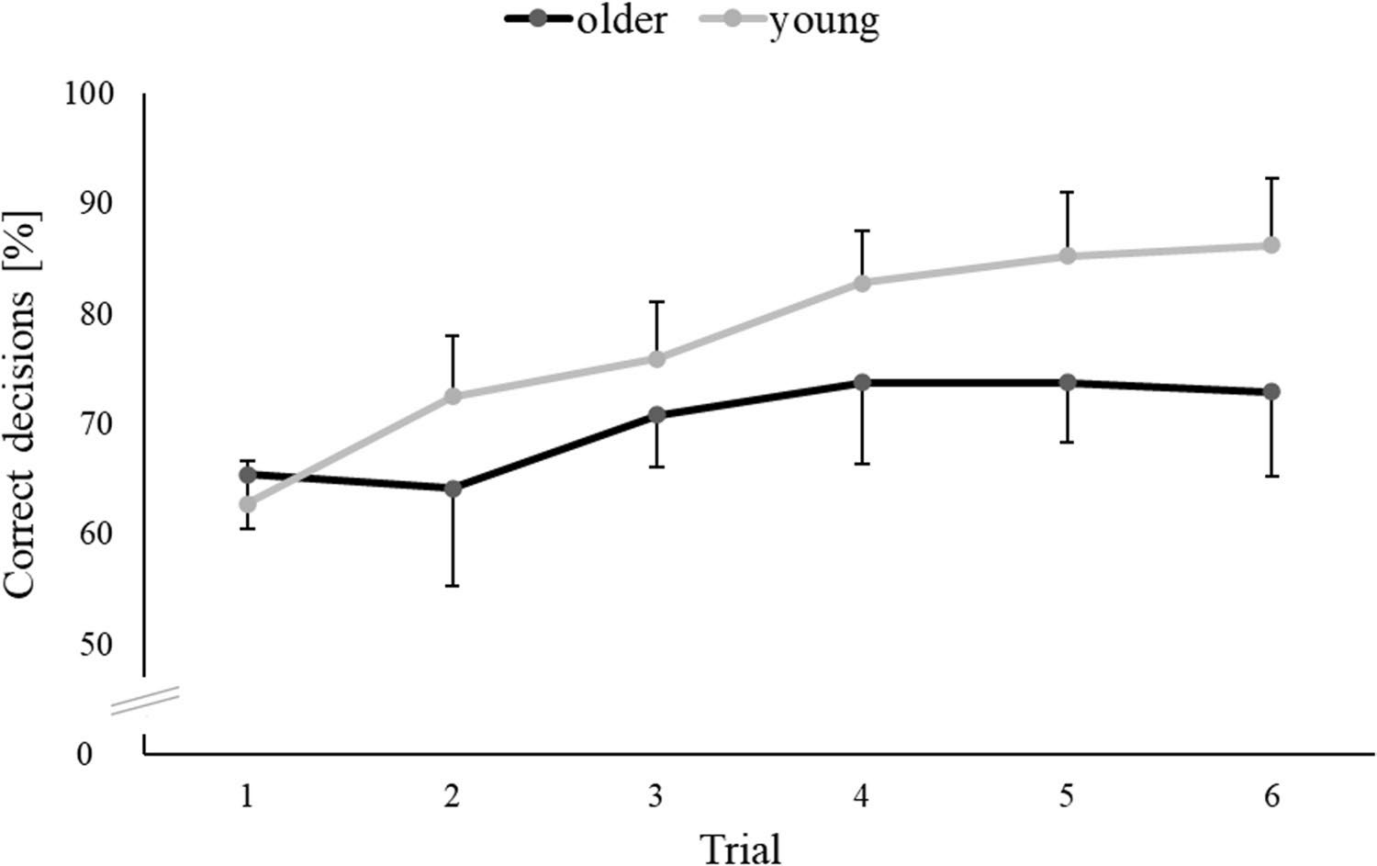
Percentage of correct decisions (PercCorDir) in the route recall test. Data are plotted separately for each trial and for either age group. Symbols represent across-participant means, and error bars are the pertinent between-participant standard deviations

Figure 4 illustrates that route recall accuracy (PercCorDir) tended to increase from trial to trial, more so in young than in older persons. Accordingly, analysis of variance for PercCorDir yielded significance of Age (*F*(1, 34) = 6.527; *p* = 0.015; *ŋ*^2^ = 0.161), Trial (*F*(3.884, 132.059) = 17.238); *p* < 0.001; *ŋ*^2^ = 0.336), and Age * Trial (*F*(3.884, 132.059) = 2.984; *p* = 0.022; *ŋ*^2^ = 0.081).

**Fig. 5:**
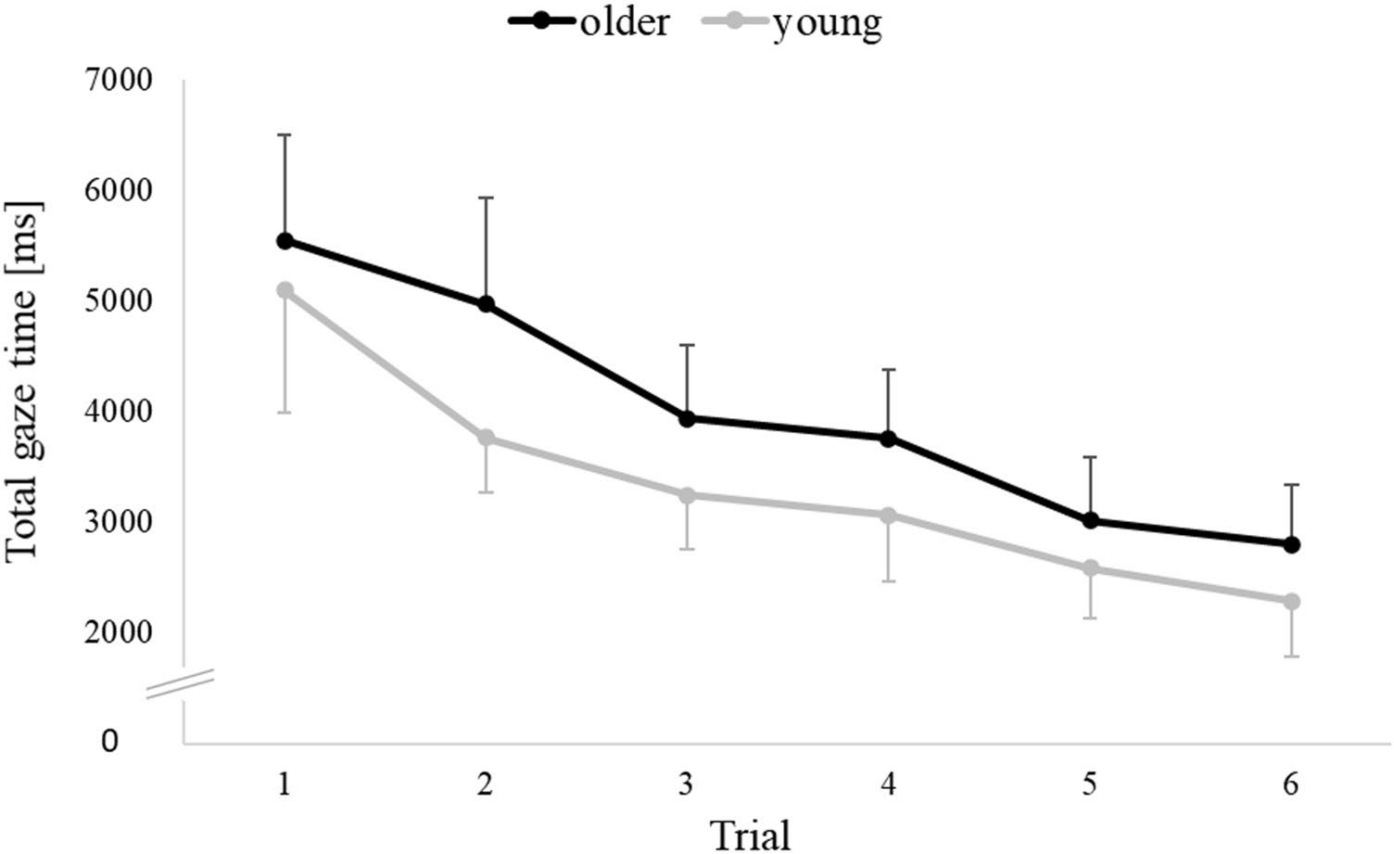
Total gaze time at a screenshot (TotGazeT) in the route recall test. Data are plotted as in Fig. 4

**Fig. 6:**
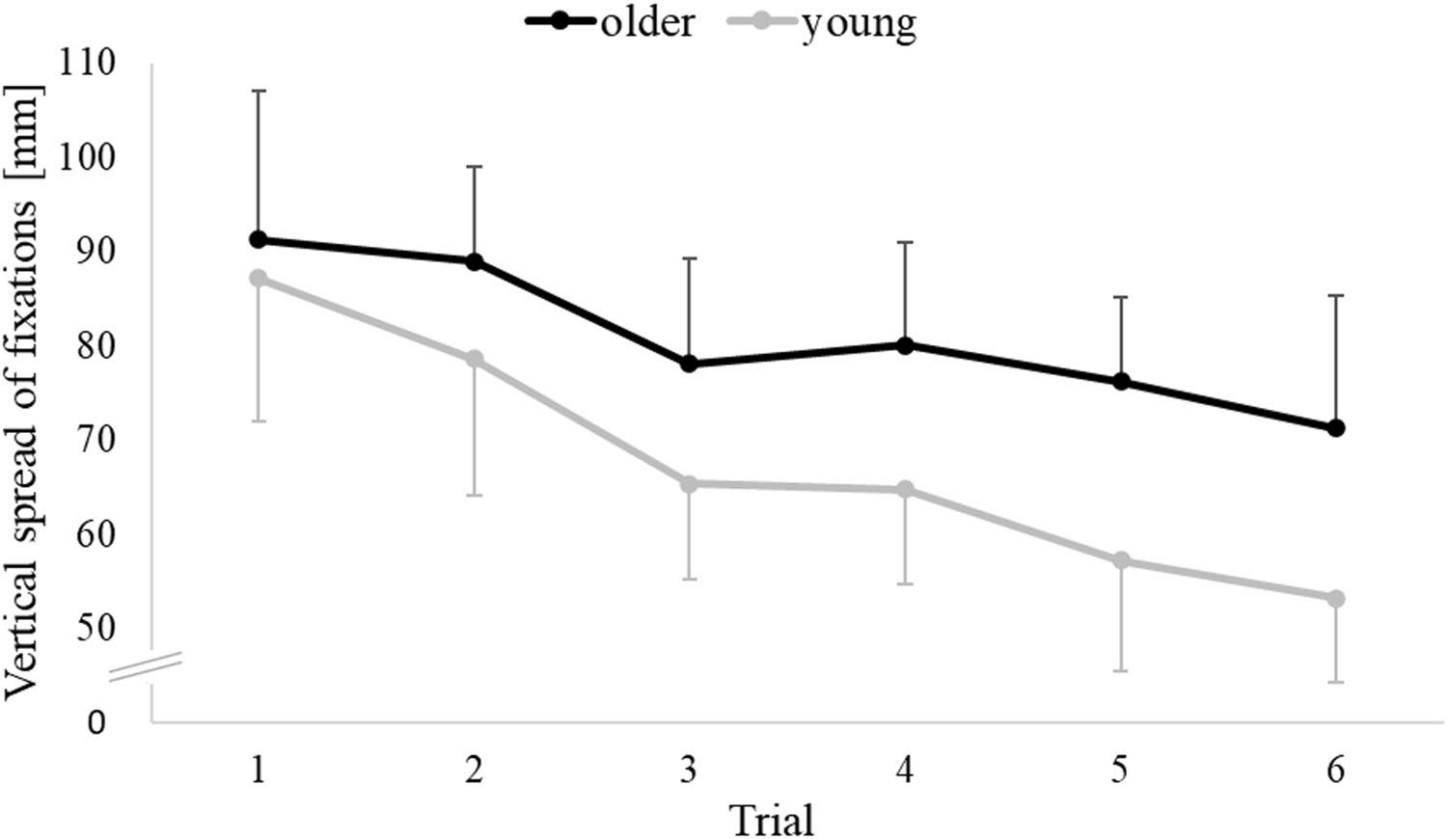
Vertical scatter of fixations at a screenshot (VertScatFix) in the route recall test. Data are plotted as in Fig. 4

Of 10,368 registered gaze scores (12 screenshots * 6 trials * 36 participants * 4 parameters), 1018 or 9.8% were invalid and had to be imputed. The multivariate general linear model yielded significance of Age (*F*(4, 31) = 5.548; *p* = 0.002; *ŋ*^2^ = 0.417) and of Trial (*F*(3.24, 110.28) = 5.076; *p* < 0.001; *ŋ*^2^ = 0.871), but no significance of Age * Trial (*F*(3.24, 110.28) = 1.114; *p* = 0.464; *ŋ*^2^ = 0.598). Post hoc decomposition of the factor Age yielded no significance for the predictors TotGazeT (*p* = 0.088), MeanFixT (*p* = 0.411), HorScatFix (*p* = 0.041) or VertScatFix (*p* = 0.033); note that the significance threshold was set to *p* < 0.01 for gaze data, because of the imputation procedure (cf. “Methods” section). Post hoc decomposition of the factor Trial yielded no significance for the predictors MeanFixT and HorScatFix; their mean ± standard deviation was 422.86 ± 122.78 ms and 191.02 ± 47.86 mm, respectively. However, post hoc decomposition of the factor Trial yielded significance for the dependent variables TotGazeT and VertScatFix, as illustrated in Figs. 5 and 6. In post hoc tests for TotGazeT, trial 1 differed from trial 2 to 6 (all *p* < 0.001), trial 2 differed from trial 3 (*p* < 0.01) and 4 (*p* < 0.001), trial 3 differed from trial 5 (*p* < 0.01) and 6 (*p* < 0.001), and trial 4 differed from trial 6 (*p* < 0.001). In post hoc tests for VertScatFix, trial 1 differed from trial 3, 5, 6 (all *p* < 0.001) and 4 (*p* < 0.01), and trial 2 differed from trial 5 (*p* < 0.01) and 6 (*p* < 0.001). In sum, we found robust evidence that TotGazeT and VertScatFix decreased consistently from trial to trial, but we found no robust evidence that gaze parameters or their trial-to-trial change were age-dependent.

The analysis of gaze parameter IncidFixChosen is detailed in the Appendix. In short, IncidFixChosen was significantly larger in older compared to young persons, and it did not change consistently from trial to trial in either age group.

Multiple linear regression with the dependent variable ‘PercCorDir on the last trial’, and with the predictors Direction Sequence Test, Landmark Sequence Test and ‘calendric age’, just missed statistical significance (*R* = 0.453, *F*(3,32) = 2.753, *p* = 0.058). Accordingly, all partial effects were non-significant (Direction Sequence Test: *F*(1,32) = 1.456; *p* = 0.236; *η*^2^ = 0.044; Landmark Sequence Test: *F*(1,32) = 0.245; *p* = 0.623; *η*^2^ = 0.008; age: *F*(1,32) = 0.868; *p* = 0.358; *η*^2^ = 0.026). Means and standard deviations were 9.528 ± 1.828 for ‘PercCorDir on the last trial’, 6.278 ± 2.943 for the Direction Sequence Test, and 4.427 ± 1.797 for the Landmark Sequence Test.

The general linear model with the dependent variable Slope-PercCorDir and with the predictors Slope-TotGazeT, Slope-MeanFixT, Slope-HorScatFix, Slope-VertScatFix, and ‘calendric age’ yielded significance for the variables Slope-TotGazeT (*t*(1,32) = − 2.8600, *p* = 0.007, *η*^2^ = 0.238), Slope-HorScatFix (*t*(1,32) = 2.8158, *p* = 0.008, *η*^2^ = 0.171), and ‘calendric age’ (*t*(1,23) = − 3.2218, *p* = 0.003, *η*^2^ = 0.201). Thus, higher positive scores on slope-PercCorDir corresponded to higher negative scores on slope-TotGazeT, and lower positive scores on slope-PercCorDir corresponded to lower negative scores on slope-TotGazeT. Furthermore, higher positive scores on slope-PercCorDir corresponded to positive scores on slope-HorScatFix, and lower positive scores on slope-PercCorDir corresponded to negative scores on slope-HorScatFix. In other words, good wayfinders gradually increased the horizontal scatter of fixations with practice, while poor wayfinders gradually decreased it. Finally, higher positive scores on slope-PercCorDir were associated with younger age.

The general linear model with the dependent variable Slope-PercCorDir, and with the predictors sex, Self-efficacy Scale, Mobility I, Mobility II, Visual Patterns Test, and ‘calendric age’, yielded significance only for ‘calendric age’ (*F*(1,34) = 5.816, *p* < 0.05, *η*^2^ = 0.146). Means ± standard deviations were 2.296 ± 1.181 for Self-efficacy Scale, 1.444 ± 0.773 for Mobility I, 1.056 ± 0.583 for Mobility II, and 21.722 ± 7.577 for the Visual Patterns Test.

## Discussion

We investigated the relationship between wayfinding practice and visuo-spatial attention, which we operationalized as gaze direction. Participants had to find their way along three prescribed routes in a virtual city; the routes covered a total of 30 intersections to avoid ceiling effects, and were walked on six trials to provide enough opportunity for practice. Each trial included a route recall test where participants saw screenshots of intersections from the wayfinding task, and had to decide which way to proceed; behavioral and gaze data were registered during this test.

Wayfinding accuracy of our participants (PercCorDir) improved from trial to trial, in accordance with earlier work (de Condappa and Wiener, 2016; Hartmeyer et al., 2017; Hilton et al., 2020; Wiener et al., 2011). This improvement was more pronounced in young than in older persons, in accordance with one earlier study (Grzeschik et al., 2019) but in disagreement with two other studies where no such age dependence was observed (Hartmeyer et al., 2017; Hilton et al., 2020). We attribute this discrepancy between studies to the different amounts of practice provided: age dependence was reported by studies that administered up to five trials (Grzeschik et al., 2019) or six trials (present data), but not in studies that administered two or three trials (Hartmeyer et al., 2017; Hilton et al., 2020). It therefore is conceivable that age-related differences reach statistical significance only after more than three trials, because those differences gradually increased from trial to trial (cf. Fig. 4). Indeed, an exploratory re-analysis of our PercCorDir data yielded no significance for the ANOVA term ‘Trial * Age’ when only the initial two or three trials were considered.

Our participants’ total gaze time (TotGazeT) decreased from trial to trial, in accordance with an earlier study (Wiener et al., 2011). Mean fixation time (MeanFixT) did not change consistently from trial to trial, also in accordance with an earlier study (Hilton et al., 2020).

The scatter of fixations decreased from trial to trial in the vertical dimension (VertScatFix), but not in the horizontal dimension (HorScatFix). We are not aware of earlier research that analysed VertScatFix and/or HorScatFix across trials. However, one study quantified scatter by a dimensionless metric, and found no trial-to-trial changes (Hilton et al., 2020). This possible disagreement between studies could be related to different amounts of practice, six trials in our study versus only two trials in Hilton et al., (2020). Indeed, re-analysis of our VertScatFix data yielded no significance for the ANOVA term ‘Trial’ when only the initial two trials were considered.

Finally, the incidence of fixations to the chosen side (IncidFixChosen) did not change significantly from trial to trial in our participants; we are not aware of earlier research that evaluated IncidFixChosen trial by trial.

Summing up, trial-to-trial changes of gaze parameters are not always consistent across studies, and this discrepancy could be related to the available amount of practice. We interpret the pattern of findings in our study as evidence for a practice-related change of visuo-spatial attention: as participants gained wayfinding experience, they reduced their attentional focus to the relevant screenshot area (hence a decrease of VertScatFix), reduced the total time in which their attention dwelled on the rejected side of the screenshot (hence a decrease of TotGazeT), but maintained the total time in which their attention dwelled on the chosen side (hence no change of IncidFixChosen).

Trial-to-trial changes of TotGazeT, MeanFixT, HorScatFix, and VertScatFix were not significantly age-dependent in our study, but IncidFixChosen increased in older age. The findings regarding MeanFixT, TotGazeT, and IncidFixChosen are in accordance with earlier work (Hilton et al., 2020; Grzeschik et al., 2019). The age-related increase of IncidFixChosen could reflect older persons’ more conservative decision-making (Ratcliff et al., 2004): they looked longer on the subsequently chosen side, possibly because they required a higher level of certainty before making a decision. Future research should scrutinize whether this presumed conservative decision-making might explain older persons’ poorer wayfinding performance. One feasible approach would be to modify the experimental design such that much more fixation points are registered per trial: it would then be possible to calculate IncidFixChosen separately for each person, and then to correlate it with Slope-PercCorDir.

Our data suggest that good wayfinders are not necessarily good at memorizing direction sequences and landmark sequences, since wayfinding accuracy (PercCorDir) on the last trial correlated little with scores on the Landmark Sequence Test and on the Direction Sequence Test. Perhaps, good wayfinders are good at associating landmarks with directions, since such associations are thought to play a role for wayfinding (Siegel and White, 1975; Waller and Lippa, 2007). Alternatively, good wayfinders may be particularly proficient at acquiring survey knowledge (i.e., at forming a mental representation of the environment), since survey knowledge is also thought to be involved in wayfinding (Lynch, 1960; Siegel and White, 1975). However, the latter interpretation would only hold if the Landmark Sequence Test and the Direction Sequence Test were largely independent of survey knowledge, which remains to be shown. Thus, further research is needed to find out what makes a good wayfinder in tasks like the present one.

Trial-to-trial improvement of wayfinding performance (Slope-PercCorDir) was related to calendric age, which replicates the significant ANOVA effect of Trial * Age on PercCorDir. More importantly, higher Slope-PercCorDir was associated with a faster decrease of total viewing time (Slope-TotGazeT), and with an increase rather than decrease of the horizontal gaze scatter (Slope-HorScatFix). In accordance with our above reasoning, this pattern of results indicates that the beneficial effects of practice on visuo-spatial attention were more pronounced in good than in poor wayfinders.

We found no significant relationship between Slope-PercCorDir and the potential predictors sex, Self-efficacy Scale, Mobility I, Mobility II, and Visual Patterns Test. It is a limitation of our study that we did not include other cognitive predictors besides visuo-spatial skill (i.e., the Visual Pattern Test). Indeed, Grzeschik et al., (2019) report a significant correlation between wayfinding performance and *verbal memory* in older, but not in young persons; this correlation possibly reflects verbal encoding of landmark–direction associations (Meilinger et al., 2008). Furthermore, Wolbers and Hegarty (2010) found participants’ wayfinding performance to correlate with the ability for *perspective taking*. Another group of promising predictors are *executive functions*: wayfinding involves executive functions such as planning, decision-making, and uncertainty resolution (Moffat et al., 2006; Wolbers and Hegarty, 2010), and executive functions are known to decay in older age (cf. meta-analyses by Bopp and Verhaeghen, 2018; Verhaeghen and Cerella, 2002).

In summary, we interpret our findings as evidence for a practice-related change of visuo-spatial attention. We posit that, as our participants gained wayfinding experience, they gradually narrowed their attentional focus to the most relevant screenshot area, reduced the total time in which their attention dwelled on the rejected side of the screenshot, but maintained the total time in which their attention dwelled on the chosen side. Practice-related changes of visuo-spatial attention were more pronounced in good wayfinders than in poor wayfinders, but they did not differ significantly between age groups. These findings indicate that wayfinding performance is associated with the dynamics of visuo-spatial attention, and that age-related deficits of wayfinding performance may not necessarily be associated with the dynamics of visuo-spatial attention. However, age-related deficits may be associated with more conservative decision-making: older persons’ attention dwelled longer on the chosen side before they made their choice.

## Figures and Tables

**Fig. 7:**
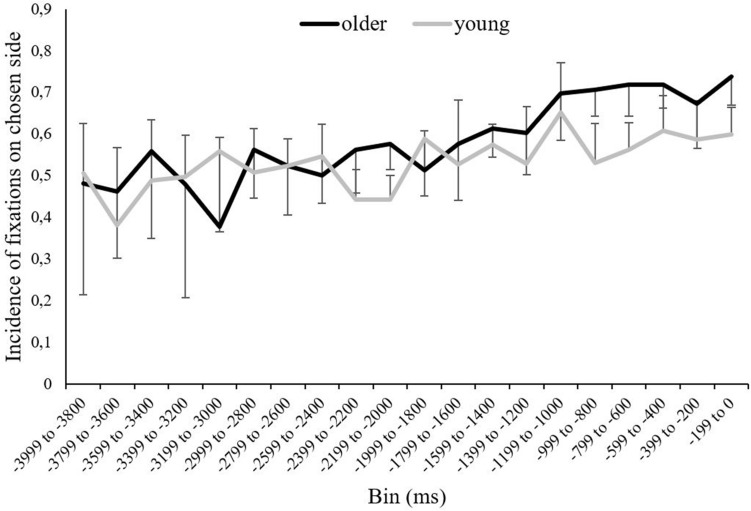
Incidence of fixations on the subsequently chosen side of the intersection (IncidFixChosen) in the two age groups. Scores were calculated for twenty 200 ms bins before the participants’ response. Data points represent mean IncidFixChosen across participants and trials, and error bars are the pertinent standard deviations

## Data Availability

The datasets generated during and/or analysed during the current study are available from the corresponding author on reasonable request.

## Appendix: Incidence of fixations on the chosen side (IncidFixChosen)

A first overview of our data revealed that the *incidence of fixations on the chosen side* began to rise beyond the chance level about 2000 ms before participants pressed the response key. We therefore decided to quantify IncidFixChosen within the last 4000 ms before the response, such that it would begin to rise after about half of the analysed time interval. We subdivided this interval into 20 bins of 200 ms width, and calculated IncidFixChosen separately for each bin but jointly across all participants from a given age group. This was a compromise, to ensure a reasonably small bin width as well as a reasonably large number of data points per bin (minimum of 46 data points).

We determined whether participants looked to the left or to the right with respect to a screenshot-specific reference direction, not with respect to the midline of the computer monitor. This was done for two reasons. First, screenshots were taken from a similar perspective as participants had adopted on the treadmill, and the displayed intersections were therefore not centred about the midline of the computer monitor; they rather were shifted slightly sideways, depending on the particular screenshot (cf. Fig. 3, where the intersection is shifted somewhat to the right). Second, some screenshots included realistic props such as bus stops or parked bicycles, which were likely to attract participants’ gaze and thus to shift it to the left or right even before IncidFixChosen began to rise. We therefore decided to calculate IncidFixChosen with respect to a screenshot-specific reference, defined as the median fixation position during the first five bins, i.e., 4000–3000 ms before participants pressed the response key.

IncidFixChosen was calculated only from screenshots, which called for a left or a right turn, and there only from trials on which participants chose the correct turn. Specifically, we calculated separately for each bin, trial and age group:

A1 $$\mathrm{IncidFixChosen}= \frac{\mathrm{number of fixations to the subsequently chosen side}}{\mathrm{number of fixations to either side}}.$$

As Fig. 7 illustrates, IncidFixChosen was about 0.5 over the first 8–10 bins, i.e., participants looked equally often to the left and to the right of the screenshot-specific reference direction. IncidFixChosen then increased, more so in older than in young persons, and established more or less a plateau over the final six bins. The level of this plateau (mean ± standard deviation of the final six bins) was 0.590 ± 0.086 in young persons and 0.709 ± 0.085 in older persons, which is a statistically significant age difference (*F*(1,70) = 34.678; *p* < 0.0001; *ŋ*^2^ = 0.331).

To find out whether IncidFixChosen changed consistently from trial to trial, we sorted the scores not by bin, as in Fig. 7, but rather by trial. We reasoned that any trial-to-trial changes should be most pronounced during the plateau phase, and we therefore limited our subsequent analysis to IncidFixChosen from the six plateau bins. Thus, in other words, each value of the variable “trial number” was combined with six scores of the variable “IncidFixChosen”, one for each plateau bin. Using these data, we found that the relationship between the predictor “trial number” and the dependent variable “IncidFixChosen” was not significant, neither in young persons (*F*(1,4) = 0.604; *p* = 0.480; *ŋ*^2^ = 0.131) nor in older persons (*F*(1,4) = 3.494; *p* = 0.134; *ŋ*^2^ = 0.466). We thus have no evidence for a consistent trial-to-trial change of IncidFixChosen.
